# Antibiotics prescribing practices in oral implantology among jordanian dentists. A cross sectional, observational study

**DOI:** 10.1186/1756-0500-4-266

**Published:** 2011-07-28

**Authors:** Ashraf E AbuKaraky, Khaldoon Abu Afifeh, Adel A Khatib, Nadiajda O Khdairi, Hanan M Habarneh, Waleed KH Ahmad, Ahmad AS Hamdan, Faleh A Sawair

**Affiliations:** 1 Department of Oral and Maxillofacial Surgery, Oral Medicine, Oral Pathology, and Periodontology, Faculty of Dentistry, The University of Jordan, Amman, Jordan; 2 Dental Department, The Islamic Hospital, Amman, Jordan; 3 Faculty of Dentistry, The University of Jordan, Amman, Jordan

**Keywords:** Antibiotics, Dental Implants, Cross Sectional Study

## Abstract

**Background:**

In oral implantology, there is no consensus on the most appropriate regimen for antibiotics prescribing, the decision to prescribe antibiotic is usually based on procedure, patient and clinician related factors. The aim of this study was to investigate the rationale of antibiotic prescribing among Jordanian clinicians who practice oral implantology.

**Findings:**

The target sample for the study was the 250 Jordan Dental Implant Group members. A five page questionnaire contained 41 questions, both closed and open questions were used to collect data. Statistical analysis was performed using SPSS Windows 16.0 (SPSS Inc., Chicago, IL, USA). Descriptive statistics were generated.

The response rate was (70.4%) 176/250. Mean age was 37.2 yrs, 49.4% always prescribe antibiotics mainly oral amoxicillin and amoxicillin with clavulinic acid. Antibiotics prescribing increased with flap raising, multiple implants and sinus or bone augmentation. Patient medical condition, periodontitis and oral hygiene were the most important clinical factors in antibiotic prescribing, non-clinical factors were; reading scientific materials, courses and lectures, knowledge gained during training, and the effectiveness and previous experience with the drug.

**Conclusions:**

Wide variations in antibiotics types, routes, dose and duration of administration were found. Recommendations on antibiotic prescribing are needed to prevent antibiotic overprescribing and misuse.

## Introduction

The practice of oral implantology has been expanding widely over the last few decades, and more patients and dental practitioners are showing interest in this field [1,2]. High success rates reported in oral implantology but failure which may have a devastating effect on both patient and clinician still occur. Several studies have investigated causes of failure and recommended measures to reduce its chances [3-9]. Infection has been implicated as one of the main reasons behind early implant failure [10], and whereas some studies found no advantage of antibiotics in ordinary dental implants insertion [11-13], many others found the contrary [14-16]. Various antibiotic regimens have been suggested; pre-operative prophylactic single or multiple doses, post operative single or multiple doses for several days or a preoperative followed by post operative doses [17-19].

Over prescribing antibiotics has a negative results on the general health and economy, therefore the proper selection of antibiotic regimen in clinical practice has a great value ^20^. The clinician decision to prescribe an antibiotic or not for a certain procedure is usually based on several factors, some factors are procedure related; the type, site, complications, sterility and duration of the procedure [21,22], patient related; dental and medical history, drug allergies and cost [21,22] and clinician related; the clinician knowledge, experience, education and working environment [22,23]. Regulating bodies had worked on guidelines of antibiotics prescribing for several surgical and medical interventions [24-30], the guidelines aid practitioners to prescribe antibiotics only when indicated and in choosing the most effective antibiotic type and dose, thus help reducing the chances of infection and the harm of antibiotics over prescribing [31,32].

In Jordan, oral implantology is practiced in the private sector, the two University Hospitals, the Royal Military Medical Services Hospitals and recently in some of the public hospitals. Jordan is a Middle Eastern country with a population of around 6 millions served by around 7000 dentists. Although it is not obligatory, most dentists who practice oral implantology in Jordan are members of the Jordan Dental Implant Group (JDIG). It had been shown that the Jordanian general dental practitioners (GDPs) inappropriately prescribed antibiotics and poorly adhered to recommended guidelines for optimum dosage and course duration [34]. No previous studies had investigated the antibiotic practice among Jordanian dentists who practice oral implantology, and up to our knowledge no other published studies had investigated this field in other countries.

The aim of this study was to investigate the rational of antibiotic prescription among Jordanian clinicians who practice oral implantology, and to investigate the influence of the procedure, patient and clinician factors on the selection of the type, dose, duration and method of administration of the antibiotic.

## Findings

### Subjects and Methods

The study was an observational study based on information collected from dentists who are members in the Jordan Dental Implant Group, therefore no ethical approval was obtained and the participants were not consented for participation in the study. The target sample was the JDIG members, as most of the Jordanian dentists who practice oral implantology are JDIG members and JGDI represent all sectors that provide the oral implantology service in Jordan. The exact number of dentists who practice oral implantology is not known but it is not expected to exceed significantly the number of the JDIG members which is currently 250, 137 are general dental practitioners (GDP) and 113 are specialists; mainly oral surgeon, prosthodontists and periodontists. A list of the names addresses and telephone numbers of the JGDI members was obtained from the JDIG, members were informed about the study by a telephone call and if agreed, a specifically designed questionnaire was sent by hand and collected a week later. Members who did not complete the questionnaire after the first week were reminded to do so by a telephone call after two weeks and again after four weeks. In case of no positive response after the third follow up call the member was considered non-responsive.

The 5 page questionnaire (additional file 1) was composed of four sections and contained 41 questions, both closed and open questions were used. The first section included questions regarding personal data, education details, work environment and level of experience in oral implantology. The second section was composed of a table with a list of the different oral implantology related procedures and questions that describe the antibiotic protocol followed in each case.

The third section included questions regarding the factors affecting decision of antibiotic prescription, the stem question was whether in all cases of dental implant insertion antibiotics are prescribed, if the answer was no, then the clinician was asked to specify whether the presence of systemic disease, oral hygiene, presence of periodontal disease, smoking and dental implant type (brand name) affect his or her decision. The fourth section was composed of two parts, the first part included open questions where clinicians are asked to write the type, dose, method of administration and duration of the antibiotic they routinely prescribe preoperatively, postoperatively or both in dental implant insertion for healthy individuals not allergic to any medications. In the second part the clinicians where asked if their choice of an antibiotic regimen was affected by the patient preference, reading scientific materials, knowledge gained during undergraduate or postgraduate training, attending courses or lectures, availability of the drug in the nearby pharmacy, advertisement, cost of the antibiotic, recommendation of other colleagues, previous experience with the drug and to specify if there were other factors.

Statistical analysis was performed using SPSS for Windows release 16.0 (SPSS Inc., Chicago, IL, USA). Descriptive statistics were generated.

### Results

Of the 250 JDIG members to whom the structured questionnaires were distributed, 176 (70.4%) returned answered questionnaires. Four of these answered questionnaires were excluded because of missing data. The demographic and professional characteristics of the 172 respondents are shown in Table 1. The mean age was 37.2 ± 8.5 years (range 23-65 years) and mean experience with dental implantology was 6 ± 4.3 years (range 1-20 years) with an average number of implant inserted of 271 ± 664 (range 1-5000 implants). Table 2 shows the antibiotics prescription choices of the 172 surveyed dentists in different dental implantology procedures in healthy patients. When asked whether they prescribe antibiotics for all dental implant insertion irrespective of the patient's medical or dental condition, 49.4% of surveyed dentists answered yes. Of those who answered no to this question, the decision was mainly affected by the presence of systemic disease (91%), periodontitis (86%), poor oral hygiene (77%), and to lesser extent smoking (48%) and the brand name of the dental implant system (14%).

**Table 1:** Demographic and professional characteristics of participating members of the Jordanian Dental Implant Society

Variable		n	%
Gender	Male	153	89.0
	Female	19	11.0
Age (years)	≤ 30	41	23.8
	31-40	85	49.4
	41-50	32	18.6
	>50	14	8.1
Level of education	Bachelor	89	51.7
	Master	66	38.4
	PhD/or equivalent	17	9.9
Specialty	GDP	96	55.8
	Oral surgeon	43	25.0
	Prosthodontist	13	7.6
	Periodontist	16	9.3
	Others	4	2.3
Country of most recent qualification	Jordan	78	45.3
	Other Arab countries	42	24.4
	Eastern Europe	22	12.8
	Asia	6	3.5
	USA/Western Europe	24	13.9
Area of employment	Private practice	135	78.5
	University hospital	10	5.8
	Military hospital	15	8.7
	Public hospital	12	7.0
Attended courses on use of antibiotics in dental implantology	Yes	102	59.3
	No	70	40.7
Read scientific material on use of antibiotics in dental implantology	Yes	137	79.7
	No	35	20.3
Experience with implants (years)	<5	80	46.5
	5-10	70	40.7
	>10	22	12.8
Number of implants inserted	<50	66	38.4
	51-100	38	22.1
	101-200	22	12.8
	>200	46	26.7

**Table 2:** Antibiotic prescription choices of 172 surveyed dentists in different dental implant procedures in healthy patients

	Antibiotic choice				
	
Procedure	I do not prescribe antibiotic for this procedure.	I prescribe only preoperative antibiotic.	I prescribe only postoperative antibiotic.	I prescribe pre- & postoperative antibiotics.	I did not do this procedure.
	n (%)	n (%)	n (%)	n (%)	n (%)
Straight forward single implant case without raising a flap (flapless).	47 (27.3)	8 (4.7)	41 (23.8)	21 (12.2)	55 (32.0)
Straight forward single implant case with raising a flap.	26 (15.1)	20 (11.6)	80 (46.5)	40 (23.3)	6 (3.5)
Straight forward multiple flapless implant case.	19 (11.0)	14 (8.1)	47 (27.3)	31 (18.0)	61 (35.5)
Straight forward multiple implant case with raising flaps.	10 (5.8)	11 (6.4)	73 (42.4)	60 (34.9)	18 (10.5)
Immediate implant placement in absence of active infection.	16 (9.3)	12 (7.0)	50 (29.1)	54 (31.4)	40 (23.3)
Immediate implant placement in presence of active infection.	4 (2.3)	9 (5.2)	21 (12.2)	75 (43.6)	63 (36.6)
Internal sinus elevation.	11 (6.4)	8 (4.7)	44 (25.6)	64 (37.2)	45 (26.2)
External sinus elevation.	3 (1.7)	7 (4.1)	38 (22.1)	62 (36.0)	62 (36.0)
Bone augmentation.	9 (5.2)	5 (2.9)	43 (25.0)	72 (41.9)	43 (25.0)
At time of gingival former (healing abutment) insertion.	127 (73.8)	4 (2.3)	7 (4.1)	8 (4.7)	26 (15.1)
At time of impression taking.	133 (77.3)	0 (0)	4 (2.3)	8 (4.7)	27 (15.7)
At time of crown delivery	132 (76.7)	0 (0)	5 (2.9)	8 (4.7)	27 (15.7)

Amoxicillin plus Clavulanic acid or amoxicillin alone were the most common preoperative and postoperative antibiotics prescribed, the routes of administration, the dosages, frequencies and the length of the courses are shown in Table 3. Other antibiotics such as Clindamycin, Lincomycin, Metronidazole, penicillin, cephalosporins, or combinations of Amoxicillin and Metronidazole, Clindamycin and Lincomycin, Amoxicillin plus Clavulanic acid and Metronidazole, Amoxicillin and Clindamycin, Amoxicillin and Erythromycin, or Amoxicillin plus Clavulanic and Clarithromycin and Azithromycin were also prescribed by some participants. The non-clinical factors influencing the choice of the antibiotic course prescribed are shown in Table 4.

**Table 3:** Examples of most commonly prescribed post-operative antibiotics by survey dentists

Pre-Operative Antibiotic (number)*	Route of administration (number)*	Dose (number)*	
Amoxicillin (32)	I.M (1)	500 mg (1)	
	Oral (31)	500 mg (15); 1000 mg (9); 2000 mg (7)	
Amoxicillin + Clavulanic acid (39)	I.M (1)	1000 mg (1)	
	Oral (38)	375 mg (1); 625 mg (23); 1000 mg (11); 2000 mg (3)	

Post-Operative Antibiotic (number)*	Dose (number)*	Daily frequency (number)*	Course duration (number)*

Amoxicillin (23)	500 mg (17)	3 times (17)	3 days (3); 4 days (2); 5 days (7); 7 days (5)
	1000 mg (6)	Once (1)	One day (1)
		3 times (1)	5 days (1)
		Twice (4)	5 days (2); 6 days (1); 10 days (1).
Amoxicillin + Clavulanic acid (49)	375 mg (1)	3 times (1)	5 days (1)
	500 mg (1)	3 times (1)	5 days (1)
	625 mg (28)	Twice (4)	7 days (4)
		3 times (24)	3 days (2); 4 days (2); 5 days (10); 6 days (2); 7 days (7); 8 days (1).
	1000 mg (17)	Once (1)	3 days (1).
		Twice (16)	1 days (1); 3 days (2); 4 days (2); 5 days (7); 6 days (1); 7 days (3).
	1250 mg (1)	Twice (1)	8 days (1)
	1875 mg (1)	Once (1)	1 day (1)

Note: all post-operative antibiotics were administered per oral route. Note: (n)* number of surveyed dentists who prescribed the antibiotic.

**Table 4:** Non-clinical factors affecting the choice of the antibiotic course prescribed by the surveyed dentists for dental implant procedures

FACTOR	YES (%)
■ Patient's preference	25

■ Reading scientific materials (e.g., books, articles, internet)	86.6

■ Knowledge gained during undergraduate or postgraduate training	86

■ Attending courses and lectures	84.9

■ Availability in the nearby pharmacy	24.4

■ Advertisement (free samples, medical representatives, ... etc)	16.3

■ Cost of the antibiotic	36

■ Recommended by other colleagues	43

■ Effectiveness and previous experience with the drug	84.3

### Discussion

The sample was representative of all the sectors that provide the oral implantology service in Jordan, and it was clear from the results that young practitioners had more interest in oral implantology and slightly less than half of the service providers were specialists with postgraduate degrees, most of them work in the private sector and many were interested in continuous education. Similar findings related to the increased interest of young generations in oral implantology had been found in other studies in Hong Kong [1] and Switzerland [34].

Antibiotics prescribing was influenced by flap raising, number of implants inserted, the timing of implant insertion in the presence or absence of active infection, bone augmentation and sinus lifting procedures. Although no evidence could be found in the literature, the participants in this study had considered the flap type, number of implants and the timing of implant insertion as factors for antibiotic prescribing. An interesting finding in the study was that 13% of the participants prescribe antibiotics at time of gingival former insertion and more interesting was that around 8% prescribe antibiotics at time of impression taking and at time of crown delivery. On the other hand, regarding timing of antibiotic prescribing, although the pre-operative administration of 2g amoxicillin had been recommended to reduce chances of implant failure [35,36], and although the benefit of postoperative administration of antibiotics if preoperative dose had been given was not confirmed [19], in our study few participants prescribe antibiotics pre-operatively compared to post-operatively and a good percentage of participants prescribe antibiotics pre and post-operatively even for simple procedures such as straight forward single implant insertion in healthy individual.

The participants in this study were nearly equally divided on whether antibiotics should always be prescribed prior to implant insertion regardless of any possible related factors. This does reflect the conflicting results and opinions found in the literature regarding oral implantology and antibiotics [11-16]. For participants who do not always prescribe antibiotics, all clinical factors taken in consideration in prescribing antibiotics except for the brand of the implant system used, can be related to increased tendency of infection due to systemic or local reasons. The relation between the implant system brand name and antibiotic prescribing was difficult to understand and the authors were unable to explain. On the other hand, with the exception of patient preference and availability in nearby pharmacy, the non-clinical factors influenced the clinician decision on prescribing and choosing antibiotics were similar to what had been found in other studies made on different medical specialities and for different medical interventions [21-23].

Wide variations in the types, routes, dose and duration of administration of antibiotics were found in the study, both amoxicillin and amoxicillin with calvulinic acid were most frequently used antibiotics pre-operatively or postoperatively. The two antibiotics are widely used in oral implantology and their role in reducing implant failure was investigated in several clinical trials [12,14-16,18,19,35,36]. Although there is no consensus yet on the most appropriate regimen for antibiotics prescribing in dental implant insertion, present evidence suggest that when compared to patients having no antibiotics, patients given a single dose of 2 g amoxicillin one hour prior to dental implant insertion might experience less implant failure [36]. Several studies found no benefit in prescribing postoperative antibiotics in patients given preoperative 2g amoxicillin [18,19], and similar early failure rates were found whether patients had a single preoperative 2g amoxicillin one hour prior to surgery or had only postoperative amoxicillin and clavulinic acid 625mgs three times daily for five days [19].

Other antibiotic regimen used included clindamycin, lincomycin, metronidazole, penicillin, cephalosporins, and some participants followed antibiotic regimens which included a combination of two or three drugs. One familiar combination which had been widely used in dentistry, was amoxicillin and metronidazole [37-39], but other combinations as Amoxicillin and Clindamycin, Amoxicillin and Erythromycin or Amoxicillin plus Clavulanic and Clarithromycin and Azithromycin might not be only unnecessary but might also be harmful for the patient and may encourage the emergence of resistant bacterial strains [31,32].

## Conclusions

Based on the result of this observational study, the main oral implantology service providers were young clinicians, many possess high level of education and had put effort to obtain knowledge mainly by reading scientific materials and attending courses. Despite this, wide variations in antibiotic prescribing practices were found, and some practices may not be justified as it might be considered as antibiotic overprescribing and more importantly might be harmful on the patient. In the authors opinion, recommendations on antibiotic prescribing are needed from international oral implantology regulating bodies based on the available evidence in the literature to help clinicians avoid antibiotic misuse, and meanwhile it might be sensible to suggest that for dental implant insertion, clinicians might give no antibiotics, a single preoperative dose or a short postoperative course.

## Competing interests

The authors declare that they have no competing interests.

## Authors' contributions

All authors had reviewed and accepted the final manuscript. AA contribution was in literature review, designing the study, data analysis, write up and editing; KAA contributed in designing the study; AK, NK, HH contributed in collecting and entering data; AH contribution was in write up and editing and FS contributed in data analysis.

## Supplementary Material

Additional file 1 **Questionnaire**. 5 page questionnaire composed of four sections and contains 41 questions. Click here for file
