# Supplementary material for: Antibiotics prescribing practices in oral implantology among jordanian dentists. A cross sectional, observational study
Source: BMC Res Notes. 2011 Jul 28;4:266. doi: 10.1186/1756-0500-4-266 (PMC3160395; doi:10.1186/1756-0500-4-266)
Supplement: Additional file 1 — Questionnaire. 5 page questionnaire composed of four sections and contains 41 questions. [file 1756-0500-4-266-S1.DOC]

| Antibiotics Prescribing Practices for Dental Implants Among Jordanian Dentists. |
| --- |
| Dentists Questionnaire |

**.**

| **Gender** | Male | | Female |  | | | | | | | | | | | |
| --- | --- | --- | --- | --- | --- | --- | --- | --- | --- | --- | --- | --- | --- | --- | --- |
| **Age (Years)** | ------------------------- | |  | | | | | | | | | | | | |
| **Level of education** | Bachelor | | Master | | Ph.D | | | | |  | | | | | |
| **Working as** | General dental practitioner | | | | | Oral and Maxillofacial Surgeon | | | | | | Prosthodontist | | Periodontist | Others |
| **Country of most recent qualification** | Jordan | Other Arab countries | | | | | | Eastern Europe | | | Asia | | | USA/Europe | Others |
| **Have you attended any course or lecture on the use of antibiotics in dental implantology within the last year?** | Yes | No | | | | | |  | | | | | | | |
| **Did you read any scientific material about the use of antibiotics in dental implantology within the last year?** | Yes | No | | | | | |  | | | | | | | |
| **Area of employment** | Private practice | University hospital | | | | | Military hospital | | Public hopital | | | | Others | |  |
| **Experience with implants** | ---------years |  | | | | | | | | | | | | | |
| **Approximately, how many implants you have already inserted during your carrier?** | ------implants |  | | | | | | | | | | | | | |

**I- In Healthy individuals, I routinely prescribe antibiotics for the following procedures:**

| **Procedure** | **I usually do not prescribe antibiotic for this procedure** | **I prescribe Preoperative antibiotic only** | **I prescribe Postoperative antibiotic only** | **I prescribe Pre- & postoperative antibiotics** | **I don't do this procedure** |
| --- | --- | --- | --- | --- | --- |
| Straight forward single implant case without raising a flap (flapless). |  |  |  |  |  |
| Straight forward single implant case with raising a flap. |  |  |  |  |  |
| Straight forward multiple implants case without raising flaps (flapless). |  |  |  |  |  |
| Straight forward multiple implants case with raising flaps. |  |  |  |  |  |
| Immediate implant placement  (Extraction & immediate implant placement)  Without presence of chronic infection at extraction site. |  |  |  |  |  |
| Immediate implant placement  (Extraction & immediate implant placement)  With presence of chronic infection at extraction site |  |  |  |  |  |
| Internal sinus elevation |  |  |  |  |  |
| External sinus elevation |  |  |  |  |  |
| Bone Augmentation |  |  |  |  |  |
| At time of gingival former (healing abutment) insertion. |  |  |  |  |  |
| At time of impression taking. |  |  |  |  |  |
| At time of crown delivery |  |  |  |  |  |
|  |  |  |  |  |  |

**2- Factors affecting decision of antibiotic prescription:**

I routinely prescribe antibiotics for all dental implant insertion cases irrespective of the patient’s medical or dental condition:

Yes No

If your answer for the above question is **No**, please indicate which of the following factors affects your decision of antibiotic prescribing for implant insertion by answering the following questions?

| **Factor** | **YES** | **NO** |
| --- | --- | --- |
| Presence of systemic diseases (e.g., Diabetes mellitus, hypertension, heart disease, …) |  |  |
| Oral hygiene of the patient |  |  |
| Periodontal disease |  |  |
| Smoking |  |  |
| The implant system (brand name) that will be used |  |  |

**III- Antibiotic details:**

**For individuals NOT allergic to any medications, please write down the details of ONE or MORE of the following antibiotic regimens that you might choose to follow in implant insertion:**

| Duration  (for how many days) | Frequency (how many times per day) | Dose | Route of administration (oral, I.M., I.V.) | Name of the antibiotic |  | |
| --- | --- | --- | --- | --- | --- | --- |
|  |  |  |  |  | 1. When you prescribe Preoperative antibiotic only | |
|  |  |  |  |  | 1. When you prescribe Postoperative antibiotic only | |
|  |  |  |  |  | Preoperative | 1. When you prescribe Pre- & postoperative antibiotics |
|  |  |  |  |  | Postoperative |

**Which of the following factors affect your choice of the antibiotic regimen**

| **FACTOR** | **YES** | **NO** |
| --- | --- | --- |

| - **Patient’s preference** |  |  |
| --- | --- | --- |
| - **Reading scientific materials (e.g., books, articles, internet)** |  |  |
| - **Knowledge gained during undergraduate or postgraduate training** |  |  |
| - **Attending courses and lectures** |  |  |
| - **Availability in the nearby pharmacy** |  |  |
| - **Advertisement (free samples, medical representatives, … etc)** |  |  |
| - **Cost of the antibiotic** |  |  |
| - **Recommended by other colleagues** |  |  |
| - **Effectiveness and previous experience with the drug** |  |  |
| **Others (please state)** |  |  |
|  |  |  |

Thank you
